# Optic nerve sheath diameter change in prediction of malignant cerebral edema in ischemic stroke: an observational study

**DOI:** 10.1186/s12883-020-01931-w

**Published:** 2020-09-22

**Authors:** Seong-Joon Lee, Mun Hee Choi, Sung Eun Lee, Ji Hyun Park, Bumhee Park, Jin Soo Lee, Ji Man Hong

**Affiliations:** 1 Department of Neurology, Ajou University School of Medicine, 164, World cup-ro, Yeongtong-gu, Suwon-si, Gyeonggi-do 16499 Republic of Korea; 2 Office of Biostatistics, Medical Research Collaborating Center, Ajou Research Institute for Innovative Medicine, Ajou University Medical Center, Suwon, Republic of Korea; 3 Department of Biomedical Informatics, Ajou University School of Medicine, Suwon, Republic of Korea

**Keywords:** Cerebral infarction, Brain edema, Optic nerve, Ultrasonography, Intracranial pressure

## Abstract

**Background:**

In acute large anterior circulation infarct patients with large core volume, we evaluated the role of optic nerve sheath diameter (ONSD) change rates in prediction of malignant progression.

**Methods:**

We performed a retrospective observational study including patients with anterior circulation acute ischemic stroke with large ischemic cores from January 2010 to October 2017. Primary outcome was defined as undergoing decompressive surgery or death due to severe cerebral edema, and termed malignant progression. Patients were divided into malignant progressors and nonprogressors. Malignant progression was divided into early progression that occurred before D1 CT, and late progression that occurred afterwards. Retrospective analysis of changes in mean ONSD/eyeball transverse diameter (ETD) ratio, and midline shifting (MLS) were evaluated on serial computed tomography (CT). Through analysis of CT at baseline, postprocedure, and at D1, the predictive ability of time based change in ONSD/ETD ratio in predicting malignant progression was evaluated.

**Results:**

A total of 58 patients were included. Nineteen (32.8%) were classified as malignant; 12 early, and 7 late progressions. In analysis of CT_postprocedure_, A 1 mm/hr. rate of change in MLS during the CT_baseline_-CT_postprocedure_ time phase lead to a 6.7 fold increased odds of early malignant progression (*p* < 0.05). For ONSD/ETD, 1%/hr. change lead to a 1.6 fold increased odds, but this association was trending (*p* = 0.249). In the CT_D1_, 1%/day change of ONSD/ETD in the CT_baseline_-CT_D1_ time phase lead to a 1.4 fold increased odds of late malignant progression (*p* = 0.021) while 1 mm/day rate of change in MLS lead to a 1.5 fold increased odds (*p* = 0.014).

**Conclusions:**

The rate of ONSD/ETD changes compared to baseline at D1 CT can be a predictor of late malignant progression along with MLS. ONSD/ETD change rates evaluated at postprocedure did not predict early malignant progression.

## Background

Early decompressive hemicraniectomy (DHC) performed in malignant middle cerebral artery (MCA) infarction patients can reduce mortality and improve functional outcomes [1]. In clinical practice, however, early DHC is not easy to apply to acute anterior circulation patients with large infarct core volumes. First, in DHC clinical trials, patients were randomized at least 12 h after onset [2], and usually did not include the patients in which intravenous (IV) thrombolysis was performed [3]. Thus patients that can be candidates for endovascular treatment may have not been included. Second, while DHC’s treatment effect in the elderly is low [4], elderly stroke patients tend to increase. Third, DHC is not without morbidity and mortality, and some argue that it results in increasing the number of surviving patients with significant functional deficits [5].

Thus imaging and clinical predictors of malignant cerebral edema have been studied to aid prediction of clinical courses. However, most of the indicators are based on initial imaging findings, such as infarct volume. On the contrary, dynamic parameters that indicate imminent herniation has not been well verified. Continuous intracranial pressure (ICP) monitoring has been indicated for such situations in severe traumatic brain injury, and an elevated ICP calls for therapeutic actions in these patients [6]. However, for malignant MCA infarction patients, invasive ICP monitoring showed inconsistent results in limited clinical reports [7, 8]. The value of recent non-invasive ICP monitoring need yet to be validated in these patients.

Optic nerve sheath diameter (ONSD) is a noninvasive method of ICP measurement that is gathering clinical interest [9]. The communication between optic nerve subarachnoid space and the chiasmatic cistern [10] results in cerebrospinal fluid (CSF) flow toward the optic nerve subarachnoid space in situations of increased ICP. Subsequent expansion of the dural sheath causes increase in the ONSD. The dural sheath 3 mm behind the globe is the preferred location for measurement, as changes are most obvious at this point [11, 12]. Dynamic changes in ONSD are thought to represent increases in ICP rather than its static values [13, 14], and thus serial bedside measurements can be practical.

Thus in the current study, in acute ischemic patients with large cores, we aimed to study the ability of computed tomography (CT)-based ONSD measurements and its time based changes in predicting malignant progression. We also aimed to compare the ONSD with midline shift (MLS), which is considered the gold standard measure for prediction of malignant edema [15] and known to be closely related to functional outcomes [16] and early mortality [17].

## Methods

### Patient enrolment

This study was performed using retrospective single center data from January, 2010 to October, 2017. From patients with intra/extracranial large artery occlusion evaluated for reperfusion treatments during this period, patients satisfying the below criteria were retrospectively included. (1) Anterior circulation large vessel occlusion with diffusion weighted image (DWI) infarct volume > 82 mL [18], (2) presentation within 6 h of onset, and (3) National Institutes of Health Stroke Scale (NIHSS) of same or over 15 [19]. NordicICE semi-automated software (NordicNeuroLab, Bergen, Norway) was used for volume measurements. Significant contralateral infarction was an exclusion criteria.

### Acute ischemic stroke management

Intravenous or intra-arterial reperfusion treatment was performed by physician’s decision. All patients at least underwent cerebral angiography. Medical methods to counter malignant cerebral edema was individualized based on a stepwise protocol [20]. Osmotherapy was performed as appropriate. Therapeutic hypothermia was performed according to previously described protocol [21].

### Classification of CT timepoints

**Fig. 1:**
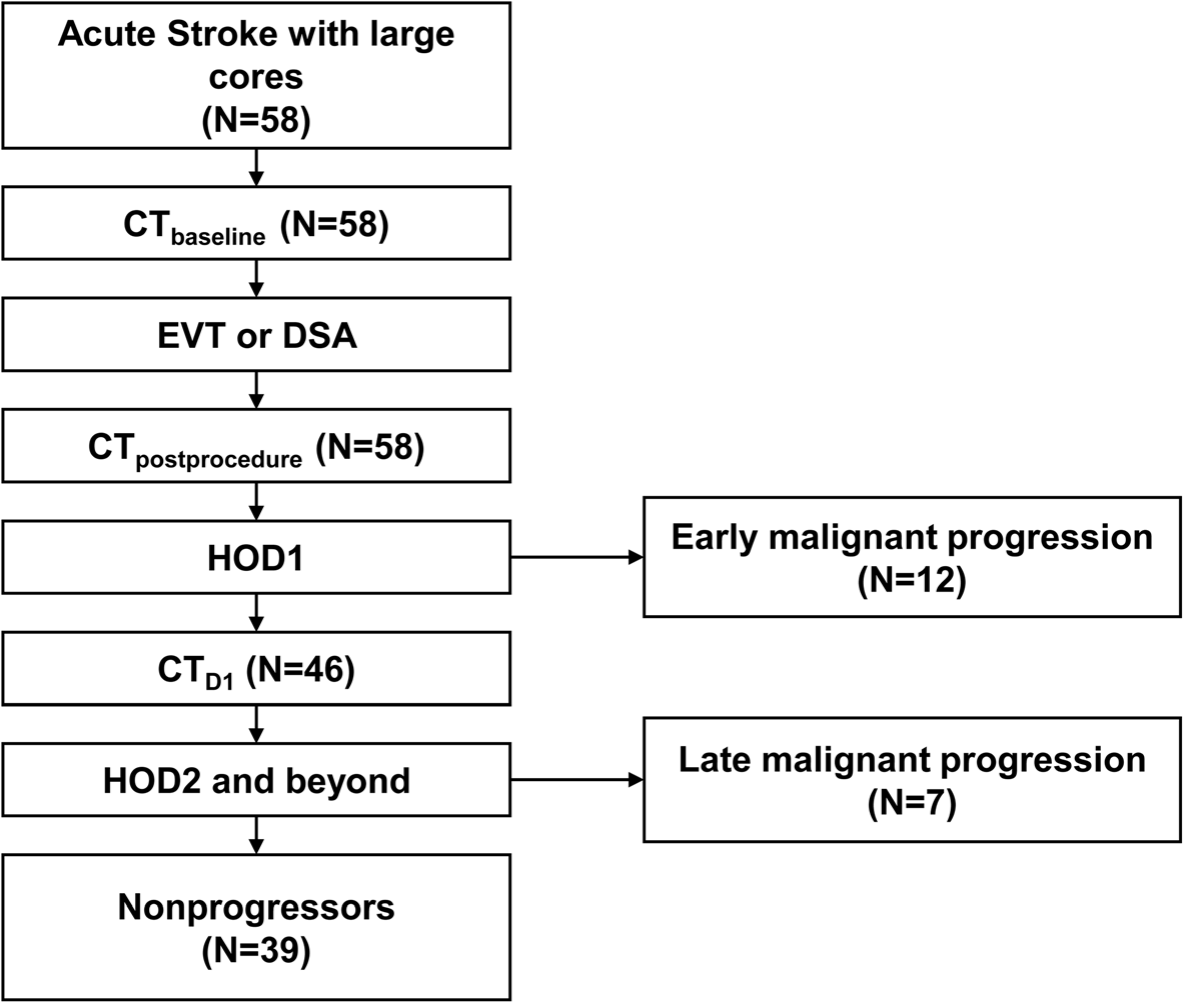
A flow chart of the included patients. Changes in CT parameters between CT_postprocedure_ and CT_baseline_ was used to predict early malignant progression (*N* = 12). In the remaining 46 patients, changes between CT_D1_ and CT_baseline_ was used to predict late malignant progression. CT, computed tomography; EVT, endovascular treatment; DSA, digital subtraction angiography; HOD, hospital day

In all patients, serial CT imaging was performed to anticipate for malignant cerebral edema. The CT scans (SOMATOM Sensation 16; SOMATOM Definition Edge [128-channel] Siemens, Erlangen, Germany) were acquired with contiguous 5-mm thick axial sections (120 kV, 270mAs). To analyse CT based factors of predicting impending herniation, CT taken at three specific time points were analysed. Baseline CT angiography upon presentation to the emergency department was classified as CT_baseline_. CT taken postprocedure after endovascular treatment or angiography was classified as CT_postprocedure_. CT angiography performed on the next day of admission was classified as CT_D1._ (Fig. 1).

### Malignant progression and decompressive hemicraniectomy

For all patients, DHC was usually performed based on clinical signs while managed with neurocritical care. During this period, patients that showed clinical signs such as altered mental status, flexor or extensor motor posturing, pupillary abnormality, repiratory pattern changes, eye movement impairments, or respiratory pattern abnormalities [22], with evidence of imminent herniation in CT was classified “malignant progressors [23].” Malignant progression was further divided into early malignant progression if it occurred after CT_postprocedure_ and before CT_D1_, and late malignant progression if it occurred after CT_D1_. DHC was performed upon consent regardless of age or whether thrombolysis was performed. Bilateral fixed pupils was a contraindication for DHC [1]. ONSD measurements were not clinically utilized.

### Measurement of CT predictors of impending herniation

**Fig. 2:**
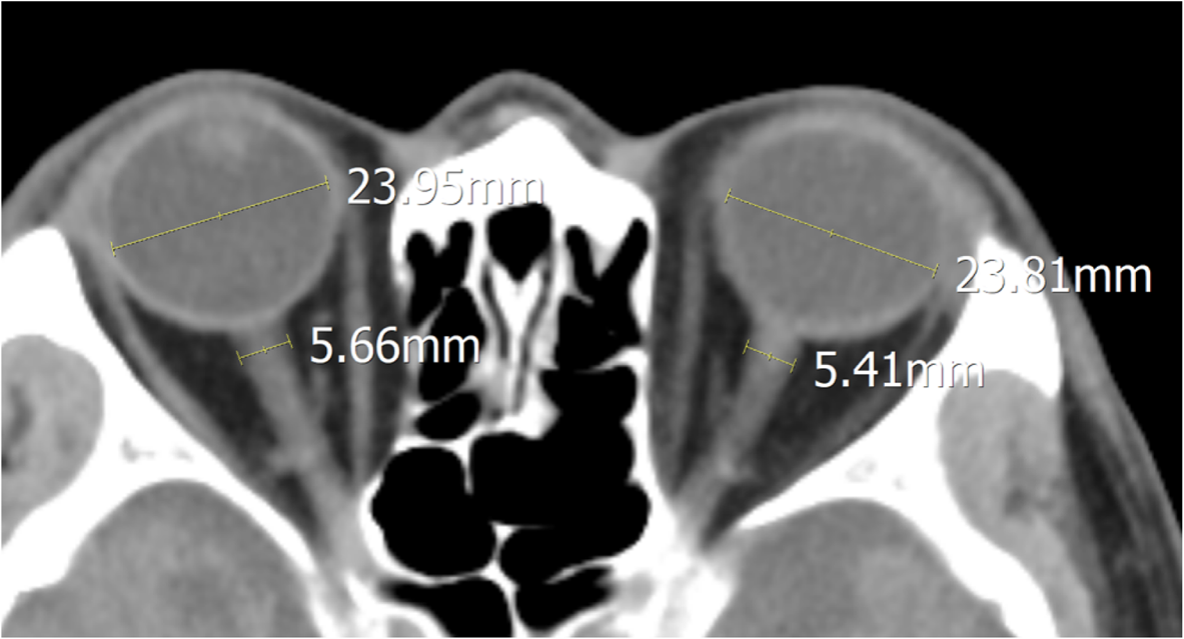
Computed tomography based measurement of ONSD and ETD. The ONSD was measured at a distance of 3 mm behind the eyeball, immediately below the sclera in a perpendicular vector with reference to the linear axis of the nerve. The ETD was defined as the maximal transverse diameter of the eyeball measured from one side of the retina to the other. ONSD, optic nerve sheath diameter; ETD, eyeball transverse diameter

The selected CT scans were evaluated using commercial image-viewing software (Picture Archiving and Communication System; Maroview 5.3 Infinitt Co., Seoul, Republic of Korea) without access to clinical information. The ONSD and eyeball transverse diameter (ETD) were measured using “chest/abdomen” window (window width 300 and window level 10) [24] with fivefold magnification. The ONSD was measured 3 mm behind the eyeball, perpendicular to the linear axis of the optic nerve [25]. The ETD was evaluated measuring the maximal transverse diameter of the eyeball from retina to retina (Fig. 2). ONSD/ETD was calculated to correct for the ETD as a confounding factor [26]. All ONSD measurement was performed bilaterally and averaged.

In the same CT scans, MLS was measured as the degree of displacement of the septum pellucidum [27] relative to the midline in the CT scans. To minimize bias, MLS measurements and ONSD measurements were performed at least 2 weeks apart. Post-procedural hemorrhagic complications were classified in accordance with the European Cooperative Acute Stroke Study criteria [28].

### Statistical analysis

The patients were divided into two groups based on malignant progression. Differences in clinical characteristics and CT profiles were compared between the two groups. Then we performed longitudinal analysis of MLS and ONSD/ETD with a linear mixed-effects model to estimate mean levels of the parameters over time within groups from baseline to D1 of follow-up, using available data. The fixed effects were CT time points, group and group-by-time interaction effect and individual was included as a random effect. Based on this preliminary analysis, imaging analysis was performed. For imaging analysis of the CT_postprocedure_ time point, both MLS changes and ONSD/ETD changes were adjusted for time by hours from CT_baseline_ to CT_postprocedure_ to account for time based changes. Multivariate analysis was further performed to confirm the clinical significance of time corrected changes in ONSD/ETD and MLS in prediction of early malignant progression. In the CT_D1_ time point, both MLS changes and ONSD/ETD changes were adjusted for time by days from CT_baseline_ to CT_D1_ to account for time based changes. Multivariate analysis was further performed to confirm the clinical significance of time corrected changes in ONSD/ETD and MLS in prediction of late malignant progression. The data are presented as the mean ± standard deviation or as the median [interquartile range]. A *P* value less than 0.05 was considered statistically significant. Statistical analyses were performed using IBM SPSS Statistics software version 25 (IBM Corp., Armonk, NY, USA) and R software, version 3.6.2. (R Foundation for Statistical Computing, Vienna, Austria).

## Results

### Clinical characteristics and initial imaging profiles (CT_baseline_)

In the overall 58 patients, early malignant progression occurred between CT_postprocedure_ and CT_D1_ (within 12 to 24 h) in 12 patients. In 7 patients, late malignant progression occurred after CT_D1_. Overall, malignant progression occurred in 19 and 39 could be managed with medical measures to reduce cerebral edema (Fig. 1). Clinical characteristics and outcomes of 19 patients classified as malignant progression are described in Table 1.

**Table 1:** Clinical characteristics of 19 patients with malignant progression and their classification process

No.	DWI volume (ml)	CT_baseline_ to malignant progression (hr)	Classification process	Decompression	3 m mRS
1	124	9.4	Pupillary changes	N	6
2	125	17.8	Pupillary changes	Y	6
3	129	21.2	Imaging based decision	Y	3
4	96	17.4	Pupillary changes	N	6
5	116	8.5	Pupillary changes	N	6
6	148	149.8	Pupillary changes	N	6
7	179	96.0	Pupillary changes	Y	5
8	218	28.9	Pupillary changes	Y	5
9	232	46.4	Pupillary changes	Y	4
10	244	13.3	Pupillary changes	N	6
11	304	19.7	Pupillary changes	Y	5
12	308	5.6	Imaging based decision	N	6
13	319	4.0	Coma	Y	6
14	364	13.2	Pupillary changes	N	6
15	391	7.8	Pupillary changes	Y	6
16	475	5.4	Imaging based decision	Y	6
17	343	5.4	Imaging based decision	Y	4
18	155	21.0	Pupillary changes	N	6
19	227	7.0	Pupillary changes	N	6

*DWI* Diffusion weighted imaging, *CT* Computed tomography, *mRS* Modified Rankin Scale

In comparison of clinical characteristics, CT angiography based occlusion location was significantly different between two groups (*p* = 0.015), with complex T type occlusions [29] much more common in the malignant progressors compared to nonprogressors (52.6% vs. 15.4%). Initial DWI volume was also significantly higher in the malignant progressors (237 ± 109 ml vs. 144 ± 43 ml, *p* = 0.002). Endovascular treatment was performed in all malignant progressors, while it was performed in 74.4% of nonprogressors (*p* = 0.015). The functional outcomes were significantly different (*p* = 0.014), with 84.2% resulting in modified Rankin Scale of 5–6 in the malignant progressors, while the percentage was 41.0% in the nonprogressors. There were no differences regarding ONSD, ONSD/ETD ratio, or MLS when the CT_baseline_ was compared (Table 2).

**Table 2:** Differences in clinical characteristics and baseline imaging profiles according to dichotomized clinical course based on malignant progression

	Malignant progressors (***n*** = 19)	Nonprogressors (***n*** = 39)	***P***-value
**Clinical characteristics**			
Sex, male	9 (47.4%)	22 (56.4%)	0.517
Age	65 ± 19	69 ± 13	0.407
Hypertension	15 (75.0%)	24 (60.0%)	0.251
Diabetes mellitus	8 (40.0%)	10 (25.0%)	0.232
Onset to door (min)	195 ± 240	173 ± 182	0.511
Hypothermia	10 (52.6%)	21 (53.8%)	0.931
Thrombolysis	12 (63.2%)	29 (74.4%)	0.379
Endovascular treatment	19 (100.0%)	29 (74.4%)	0.015
Successful reperfusion	11 (57.9%)	22 (56.4%)	0.915
Outcomes (3 month mRS)			0.014
0	0 (0.0%)	2 (5.1%)	
1	0 (0.0%)	2 (5.1%)	
2	0 (0.0%)	5 (12.8%)	
3	1 (5.3%)	4 (10.3%)	
4	2 (10.5%)	10 (25.6%)	
5	3 (15.8%)	9 (23.1)	
6	13 (68.4%)	7 (17.9%)	
**CT**_**baseline**_			
CT angiography occlusion			0.015
Complex T	10 (52.6%)	6 (15.4%)	
Simple T	4 (21.1%)	11 (28.2%)	
MCA M1	3 (15.8%)	14 (35.9%)	
Tandem	0 (0.0%)	7 (17.9%)	
Proximal ICA	1 (5.3%)	0 (0.0%)	
Dissection	1 (5.3%)	1 (2.6%)	
Initial DWI volume (ml)	237 ± 109	144 ± 43	0.002
ONSD (mm)	5.17 ± 0.55	5.37 ± 0.63	0.233
ONSD/ETD ratio	22.84 ± 2.33%	23.60 ± 2.83%	0.316
MLS (mm)	0.44 ± 0.78	0.37 ± 0.95	0.789

Values are presented as mean ± SD, number (%), or median (interquartile range)

*mRS* Modified Rankin Scale, *CT* Computed tomography, *MCA* Middle cerebral artery, *ICA* Internal carotid artery, *DWI* Diffusion weighted images, *ONSD* Optic nerve sheath diameter, *ETD* Eyeball transverse diameter, *MLS* Midline shift

### Longitudinal analysis of MLS and ONSD/ETD with a linear mixed-effects model

**Fig. 3:**
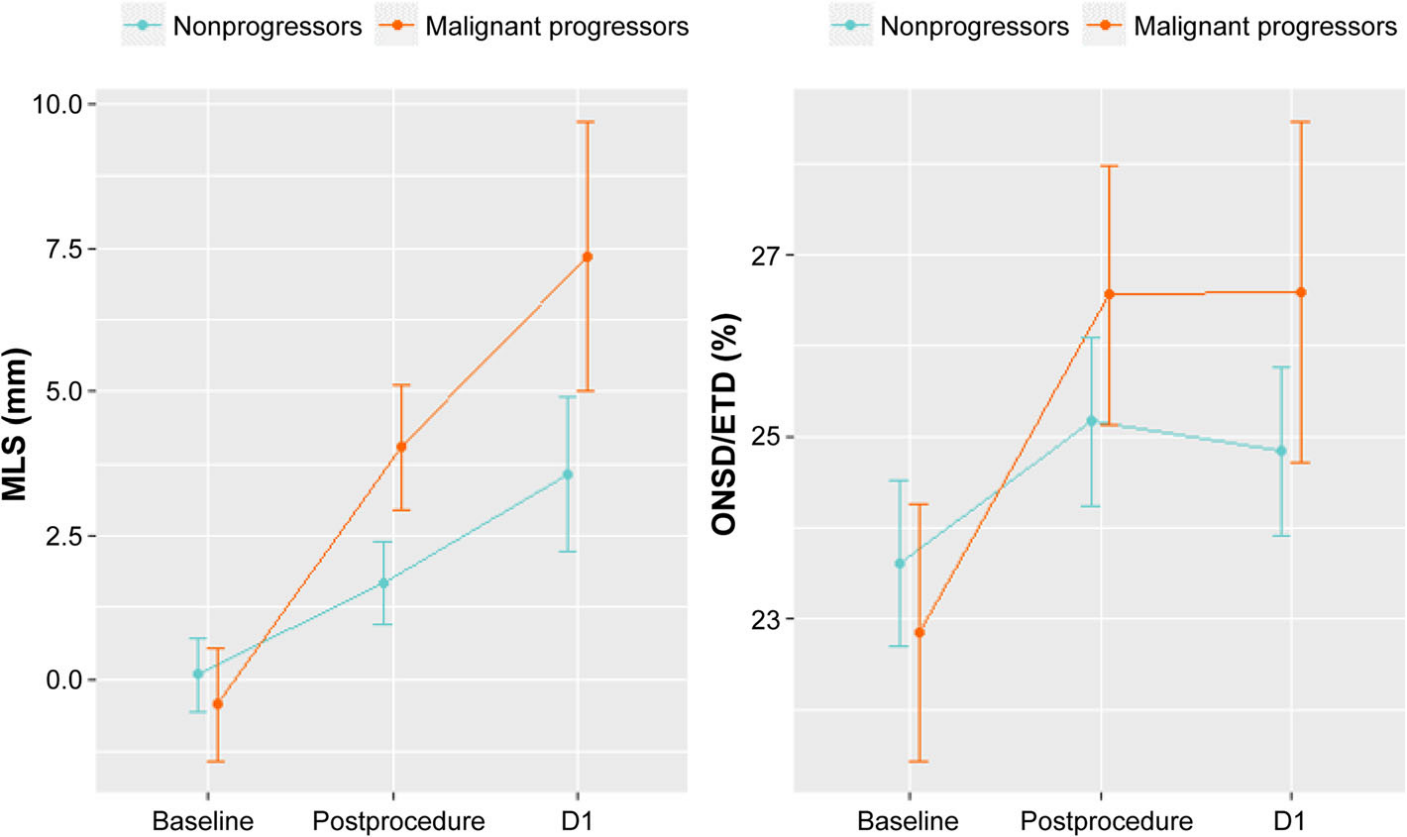
Trajectories of MLS (left panel) and ONSD/ETD (right panel) of follow-up. The time series change in MLS and ONSD/ETD, adjusted for age, sex and DWI volume, are plotted from baseline to D1. The rate of increase of MLS is constantly higher in the malignant progressors throughout the time periods. For ONSD/ETD ratio, it is increased at the postprocedure time point, and this difference is maintained. Due to a slight increase of ONSD/ETD ratio in the postprocedure time point in the nonprogressors, this difference becomes more evident at the D1 timepoint. Error bars represent 95% CIs. MLS, midline shift; ONSD, optic nerve sheath diameter; ETD, eyeball transverse diameter

When the trajectories of MLS and ONSD/ETD was compared, MLS increased at a steeper angle in the malignant progressors compared to the nonprogressors throughout the timepoints. For ONTD/ETD, it increased at a steeper angle in the malignant progressors up to postprocedure time point, and then this difference was maintained up to the D1 time point. However, a transient rise was seen in the nonprogressors at the postprocedure time point, decreasing the gap at this point (Fig. 3). In the longitudinal analysis with a linear fixed affects model, the time and group-by-time interaction effects were significant (*P* < 0.05), showing that the changes in MLS and ONSD/ETD ratio according to time points were significantly different in the nonprogressors and malignant progressors.

### Comparison of CT_postprocedure_ in prediction of early malignant progression

Early malignant progression occurred in 12 patients with a mean time from CT_baseline_ to malignant progression of 10.8 ± 7.4 h. In the CT_postprocedure_, the patients that showed early malignant progression showed significant increases in MLS (6.08 ± 4.82 mm vs. 1.69 ± 2.21 mm, *p* = 0.009), and more frequent hemorrhagic transformation (*p* < 0.001). Changes in ONSD/ETD ratio was not significantly different (3.70 ± 2.78% vs. 1.89 ± 3.37%, *p* = 0.093) (Table 3). When the changes in MLS and ONSD/ETD were normalized by time from CT_baseline_ to CT_postprocedure_, the rate of change in MLS was associated with increased odds of early malignant progression with age, sex, and initial infarct volume as covariables. A 1 mm/Hr rate of change in MLS during this time phase lead to a 6.67 fold increase in the odds of early malignant progression (*p* < 0.05). For ONSD/ETD, 1%/Hr change in this time phase lead to a 1.64 fold increase in the odds of early malignant progression, but this association was trending (*p* > 0.05) (Table 4).

**Table 3:** Comparison of optic nerve sheath diameter related parameters, midline shift, and hemorrhagic patterns on postprocedure and D1 CT images according to early and late malignant progression

**CT**_**postprocedure**_			
	**Early malignant progressors** **(*n*** **= 12)**	**Nonprogressors** **(*n*** **= 46)**	***P*-value**
CT_baseline_ to malignant progression, hr	10.8 ± 7.4		
Δ time (CT_Postprocedure_-CT_baseline_), hr	3.9 ± 1.2	4.2 ± 3.2	0.716
ONSD (mm)	5.92 ± 0.56	5.81 ± 0.71	0.599
ONSD/ETD ratio, %	26.24 ± 1.99	25.45 ± 2.9	0.382
Δ ONSD/ETD ratio, %	3.70 ± 2.78	1.89 ± 3.37	0.093
MLS (mm)	6.08 ± 4.82	1.69 ± 2.21	0.009
Hemorrhagic pattern			< 0.001
No hemorrhage	0 (0.0%)	14 (30.4%)	
HI 1	0 (0.0%)	10 (21.7%)	
HI 2	1 (8.3%)	12 (26.1%)	
PH 1	4 (33.3%)	4 (8.7%)	
PH 2	7 (58.3%)	6 (13.0%)	
**CT**_**D1**_			
	**Late malignant progressors** **(*n*** **= 7)**	**Nonprogressors** **(*n*** **= 39)**	***P*-value**
CT_baseline_ to malignant progression, hr	52.6 ± 51.5		
Δ time (CT_D1_-CT_baseline_), hr	20.0 ± 11.3	27.4 ± 11.9	0.136
ONSD (mm)	6.10 ± 0.36	5.67 ± 0.74	0.138
ONSD/ETD ratio, %	26.93 ± 2.64	24.83 ± 2.94	0.085
Δ ONSD/ETD ratio, %	3.60 ± 2.67%	1.05 ± 2.69	0.025
MLS (mm)	6.55 ± 2.87	3.64 ± 3.68	0.054
Hemorrhagic pattern			0.819
No hemorrhage	3 (42.9%)	22 (56.4%)	
HI 1	1 (14.3%)	7 (17.9%)	
HI 2	1 (14.3%)	4 (10.3%)	
PH 1	2 (28.6%)	6 (15.4%)	
PH 2	0 (0.0%)	0 (0.0%)	

Values are presented as mean ± SD, number (%), or median (interquartile range)

*CT* Computed tomography angiography, *ONSD* Optic nerve sheath diameter, *ETD* Eyeball transverse diameter, *MLS* Midline shift, *HI* Hemorrhagic infarct, *PH* Parenchymal hematoma

**Table 4:** Logistic regression models for prediction of early and late malignant progression with changes in MLS and ONSD/ETD ratio normalized by time interval

Variables	OR (95% CI)	*P*-value	Variables	OR (95% CI)	*P*-value
Early malignant progression					
Change from baseline in MLS, mm/hr. ^a^	6.67 (1.51–29.53)	0.012	Change from baseline in ONSD/ETD, %/hr. ^a^	1.64 (0.71–3.79)	0.249
Age	1.08 (0.98–1.18)	0.114	Age	1.03 (0.97–1.09)	0.379
Sex, male	0.77 (0.09–6.53)	0.814	Sex, male	0.77 (0.13–4.77)	0.783
Initial DWI volume	1.02 (1.01–1.04)	0.006	Initial DWI volume	1.02 (1.01–1.03)	0.001
Late malignant progression					
Change from baseline in MLS, mm/day ^b^	1.47 (1.08–2.01)	0.014	Change from baseline in ONSD/ETD, %/day ^b^	1.38 (1.05–1.81)	0.021
Age	0.93 (0.85–1.03)	0.178	Age	0.97 (0.9–1.04)	0.345
Sex, male	0.24 (0.02–3.74)	0.308	Sex, male	0.78 (0.11–5.82)	0.811
Initial DWI volume	1.02 (0.99–1.04)	0.136	Initial DWI volume	1.02 (1.00–1.04)	0.082

^a^Rate of change between CT_baseline_ and CT_postprocedure_

^b^Rate of change between CT_baseline_ and CT_D1_

*MLS* Midline shift, *ONSD* Optic nerve sheath diameter, *ETD* Eyeball transverse diameter, *OR* Odds ratio, *DWI* Diffusion weighted imaging

### Comparison of CT_D1_ in prediction of late malignant progression

Late malignant progression occurred in 7 patients with a mean time from CT_baseline_ to malignant progression of 52.6 ± 51.5 h. In the CT_D1_, the patients that showed early malignant progression showed significant Changes in ONSD/ETD ratio compared to baseline (3.60 ± 2.67% vs. 1.05 ± 2.69%, *p* = 0.025). However, MLS (*p* = 0.054) or hemorrhagic patterns (*p* = 0.838) were not different between the groups at this timepoint (Table 3). When the changes in MLS and ONSD/ETD were normalized by time from CT_baseline_ to CT_D1_, the rate of change from baseline to CT _D1_ in both ONSD/ETD and MLS was associated with increased odds of early malignant progression with age, sex, and initial infarct volume as co-variables. For ONSD/ETD, 1%/Day change in this time phase lead to a 1.38 fold increase in the odds of late malignant progression (*p* = 0.021). For MLS, 1 mm/Day rate of change in MLS during this time phase lead to a 1.47 fold increase in the odds of late malignant progression (*p* = 0.014). (Table 4).

## Discussion

The results of this study shows that in acute stroke patients with malignant MCA profile, early increases in ICP evaluated by ONSD/ETD increase rates at D1 can predict future malignant progression. When evaluated postprocedure, its rate of increase was not predictive of early malignant progression. Rate of changes in MLS could predict impending malignant progression both at the postprocedure and D1 time points. These dynamic markers seem to be especially valuable when evaluated in the early phase of treatment, and interpreted by evolution time.

Through utilizing time based changes of ONSD/ETD in a patient group that was primarily managed with neurocritical care with late decisions for DHC, the main result of this study shows that elevations in ONSD/ETD ratio compared to baseline within 1 day can predict malignant progression, and may call for therapeutic interventions. This is consistent with recent reports that emphasize the value of dynamic changes in ONSD [30]. Such clinical application is identical to invasive ICP monitoring in traumatic brain injury, in which elevations in ICP above 20 mmHg can predict clinical deterioration and calls for therapeutic intervention [31]. Further accumulating evidence of the value of dynamic changes in ONSD will support active use of ONSD measurements in neurocritical care.

The rate of ONSD/ETD fail to prove its significance in predicting malignant progression at the postprocedure time point. ICP is subject to changes according to changes in posture [32], or pain [33] related to acute procedures. The postprocedure period is exposed to such factors, and may have hampered the significane of ONSD/ETD at this period. This is supported by the fact that the mean trajectory of ONSD/ETD increases at the postprocedural time point and then decreases at D1 in the nonprogressors. Thus the optimal time to evaluate ICP changes compared to baseline may be when the patient is stabilized, likely at an intensive care unit setting.

Theoretically, prolonged elevations in ICP suggest failure of compensatory mechanism, resulting in decreased cerebral perfusion pressure and cerebral blood flow, increase in ischemia, and fulminant progression of herniation [34]. However, previous studies utilizing intraparenchymal ICP sensors [8] or epidural probes [7] in malignant cerebral edema suggested that pupillary abnormalities and severe brainstem compression may be present despite normal ICP values. This may possibly be due to the distance between the site of ICP probe insertion and the site of herniation, because the pressure gradient force is inversely proportional to the distance [20]. In this regard, ONSD changes may be a superior way of ICP monitoring for malignant stroke patients, for it physiologically directly represents intraventricular pressure [10], which is gold standard. Future studies with invasive ICP monitoring are needed to answer this question.

MLS is a well validated marker of malignant edema [15], and it is closely related with functional outcomes [16] and early mortality [17]. Some institutions have used a midline shift of more than 5 mm as an indicator of surgical management [8]. The rate of MLS increases according to time could predict both early and late malignant progression. However, when interpreted as an absolute value, there were no significant difference between the two groups in the D1 CT. This result shows that its evolution speed is more important than MLS as an absolute value. For example, MLS of 5 mm observed 4 h after admission and 5 mm observed 5 days after admission differs in clinical significance, and it is highly likely that the former case will need surgical intervention. To represent such temporal changes in time, variables such as infarct growth rate [35] is also being increasingly used.

The clinical role of ONSD based noninvasive measurements of ICP in ischemic stroke is increasingly gathering attention. In previous literatures, it has been shown that ONSD increases in early ischemic stroke compared to normal controls [36]. In ischemic stroke subtypes, a total anterior circulation infarct type was associated with elevations in ONSD compared to control and other stroke subtypes [36]. It has been further shown that ONSD can differentiate ischemic stroke and hemoarrhagic stroke with moderate predictive power [37]. A recent study compared the ONSD/ETD in initial CT scans of malignant MCA patients and age and gender matched controls. The authors could show that malignant MCA patients showed significantly higher ONSD/ETD ratio [38]. Such results and the current study results may advocate for CT or ultrasound based measurements of ONSD for ischemic stroke as an easily accessible measure to guide therapy.

There are some limitations to the study. First is that invasive ICP monitoring was not performed. This is due to current practice guidelines that do not recommend invasive monitoring in ischemic stroke [20]. Instead, the current results suggest that ONSD/ETD ratio measurements may be used as an alternative method for ICP measurements in these patients. However, while the association between invasive ICP values and ONSD/ETD ratio have been proven in previous studies, its correlation in ischemic stroke patients still need to be proven. Second, in evidence based practice, there are some factors that limit generalization of this retrospective study. Because a large number of patients were included before 2015, patient selection criteria for endovascular treatment was permissive. Furthermore, osmotherapy, sedation, and hypothermia are still of unproven benefit. However, malignant stroke is a rare clinical situation, and the authors think that this study results can guide tailored neurocritical management of such patients. Third, since the usual thickness for ONSD nears 5 mm, the 5 mm slice thickness CT protocol may introduce a certain margin of error due to partial volume effect [39]. Nevertheless, previous studies have shown the usefulness of ONSD measurements in 5 mm CT thickness [40], as was the result in our study. Fourth, because of its timely accessibility and utility at bedside, ultrasound based ONSD measurements may be most preferable to guide therapy in ischemic stroke patients treated with intensive medical therapy to control cerebral edema. In this regard, larger prospective studies are needed to confirm the value of serial ONSD guided managements in malignant cerebral infarcts, possibly by ultrasound at the bedside.

## Conclusion

In conclusion, in acute stroke patients with malignant infarct cores, an increase in ONSD/ETD ratio compared to baseline increases the odds of malignant progression, and may be used as a marker for emergent therapeutic interventions.

## Data Availability

The datasets used and/or analysed during the current study are available upon reasonable request to the corresponding author.

## Abbreviations

CSF: Cerebrospinal fluid
CT: Computed tomography
DHC: Decompressive hemicraniectomy
DWI: Diffusion weighted image
ETD: Eyeball transverse diameter
ICP: Intracranial pressure
MCA: Middle cerebral artery
MLS: Midline shift
NIHSS: National Institutes of Health Stroke Scale
ONSD: Optic nerve sheath diameter
